# Supplementary material for: Mechanisms governing the pioneering and redistribution capabilities of the non-classical pioneer PU.1
Source: Nat Commun. 2020 Jan 21;11:402. doi: 10.1038/s41467-019-13960-2 (PMC6972792; doi:10.1038/s41467-019-13960-2)
Supplement: Supplementary file 7 — Source data [file 41467_2019_13960_MOESM7_ESM.zip › Source_Data/FigureS2/FigureS2B_MotifScanOutput/KmeansCluster4/homerResults/motif20.similar.html]

motif20

## Information for motif20

A
C
G
T
C
T
A
G
T
C
G
A
A
G
C
T
C
G
A
T
C
T
A
G
T
C
A
G
G
T
C
A
A
G
C
T
A
G
T
C
C
G
T
A
A
G
C
T
A
T
C
G
  
Reverse Opposite:  

T
A
G
C
T
C
G
A
C
G
A
T
T
C
A
G
C
T
G
A
A
C
G
T
A
G
T
C
G
A
T
C
G
C
T
A
T
C
G
A
A
C
G
T
A
G
T
C
G
T
C
A
  

|  |  |
| --- | --- |
| p-value: | 1e-51 |
| log p-value: | -1.186e+02 |
| Information Content per bp: | 1.776 |
| Number of Target Sequences with motif | 103.0 |
| Percentage of Target Sequences with motif | 2.28% |
| Number of Background Sequences with motif | 91.1 |
| Percentage of Background Sequences with motif | 0.22% |
| Average Position of motif in Targets | 119.1 +/- 73.0bp |
| Average Position of motif in Background | 99.2 +/- 65.0bp |
| Strand Bias (log2 ratio + to - strand density) | -0.7 |
| Multiplicity (# of sites on avg that occur together) | 1.00 |
| Motif File: | file (matrix) reverse opposite |

### Similar de novo motifs found

|  |  |  |  |  |  |  |  |
| --- | --- | --- | --- | --- | --- | --- | --- |
| Rank | Match Score | Redundant Motif | P-value | log P-value | % of Targets | % of Background | Motif file |
| 1 | 0.942 | A G T C G T A C T C G A C G A T T C A G G C T A G C A T G A T C G A T C G C T A C T G A G A C T A G T C G T C A | 1e-47 | -110.273697 | 2.48% | 0.31% | motif file (matrix) |
| 2 | 0.948 | A C G T C T A G C T G A G A C T A C G T C A T G C T A G C T G A A G C T A G T C C G T A A G C T | 1e-47 | -109.344963 | 1.97% | 0.17% | motif file (matrix) |
| 3 | 0.919 | A C G T C T A G T C G A A G C T C G A T C T A G C T A G C T G A A C G T A G T C C G T A | 1e-43 | -99.939928 | 2.06% | 0.23% | motif file (matrix) |
